# Supplementary material for: Operando X-ray photoelectron spectroscopy of solid electrolyte interphase formation and evolution in Li2S-P2S5 solid-state electrolytes
Source: Nat Commun. 2018 Jun 27;9:2490. doi: 10.1038/s41467-018-04762-z (PMC6021442; doi:10.1038/s41467-018-04762-z)
Supplement: Supplementary file 1 — Supplementary Information [file 41467_2018_4762_MOESM1_ESM.docx]

­­

**Supporting Information:**

***Operando* X-Ray photoelectron spectroscopy of solid electrolyte interphase formation and evolution in Li_2_S-P_2_S_5_ solid-state electrolytes**

***K.N. Wood et.al***


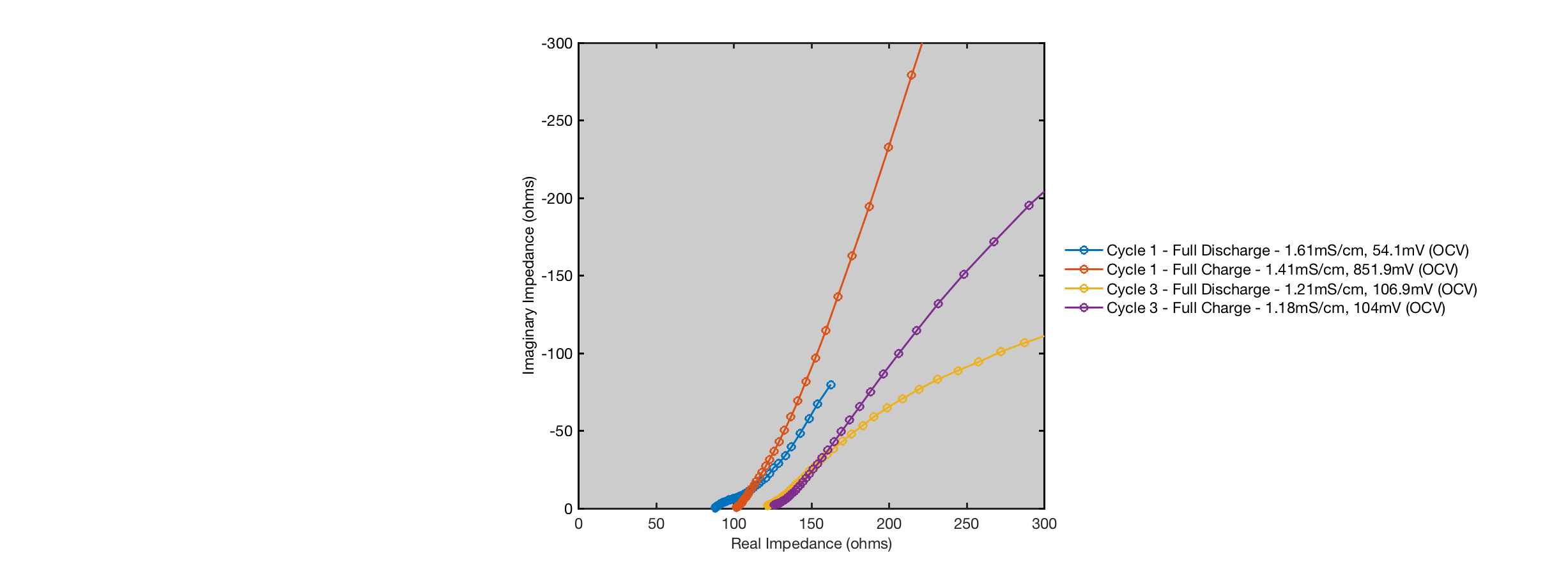


Supplementary Figure 1. EIS spectra showing the evolution of the SEI during the first three cycles of a Cu/LPS/Li coin cell.


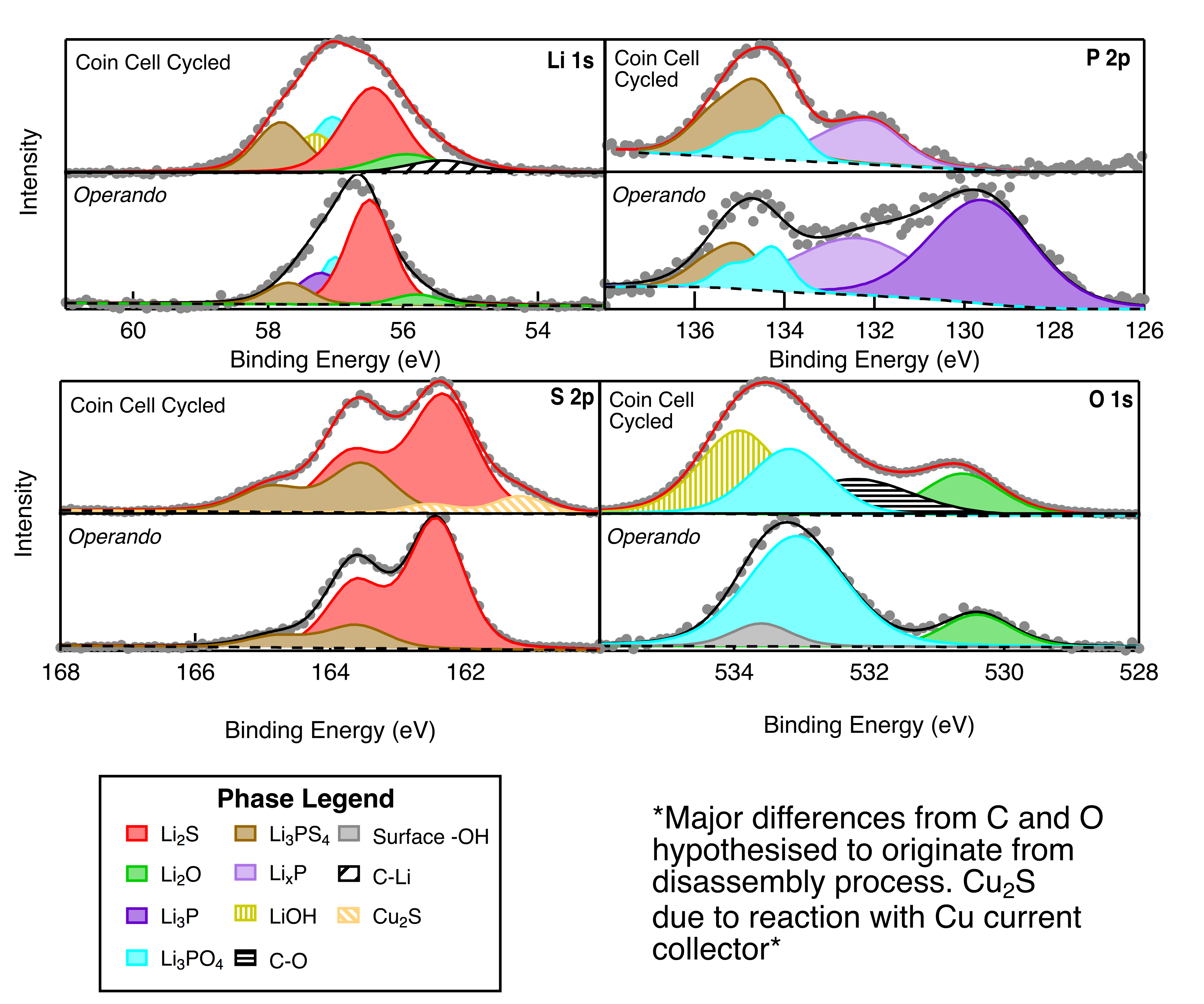


Supplementary Figure 2. XPS spectra comparing the coin cell cycled sample after 1.5 h of charging at 0.17 mA/cm^2^ and the operando XPS spectra after approximately 2 h of charge at ~0.17 mA/cm^2^.


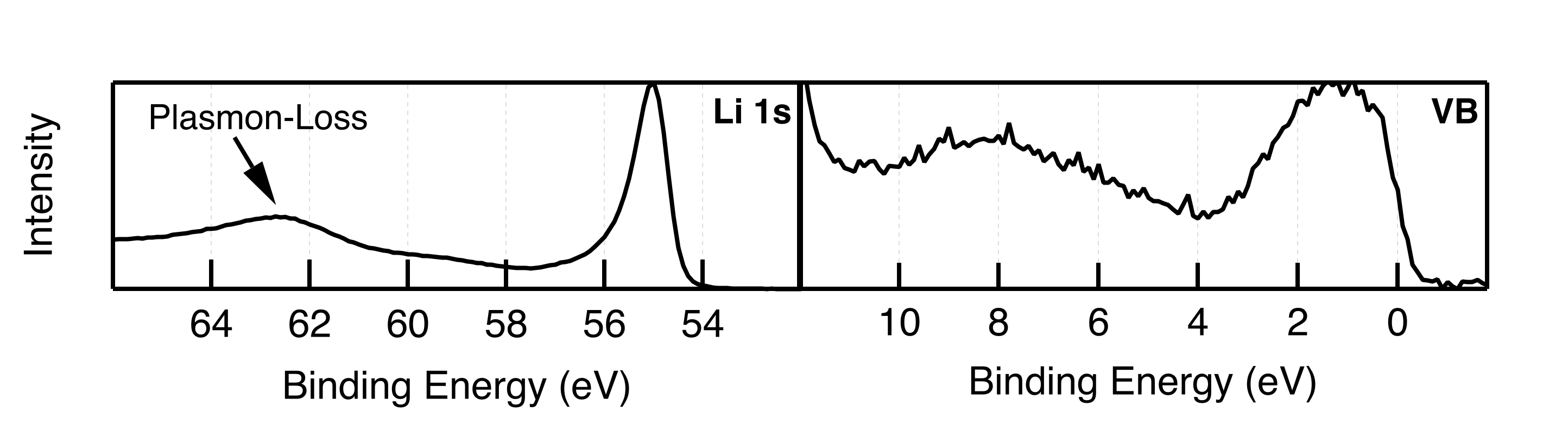


Supplementary Figure 3. Reference Li 1s and VB spectra acquired from a Li-foil sample following extensive Ar^+^-ion sputter cleaning to obtain a ‘pristine’ Li metal surface (0.5 atomic % residual oxygen). Characteristic features observed for pristine Li^0^ include: a Li 1s peak at 55.0 eV; a plasmon-loss feature at ~ 62.5 eV; and a valence-band spectrum characterized by a metallic Fermi edge at 0.0 eV


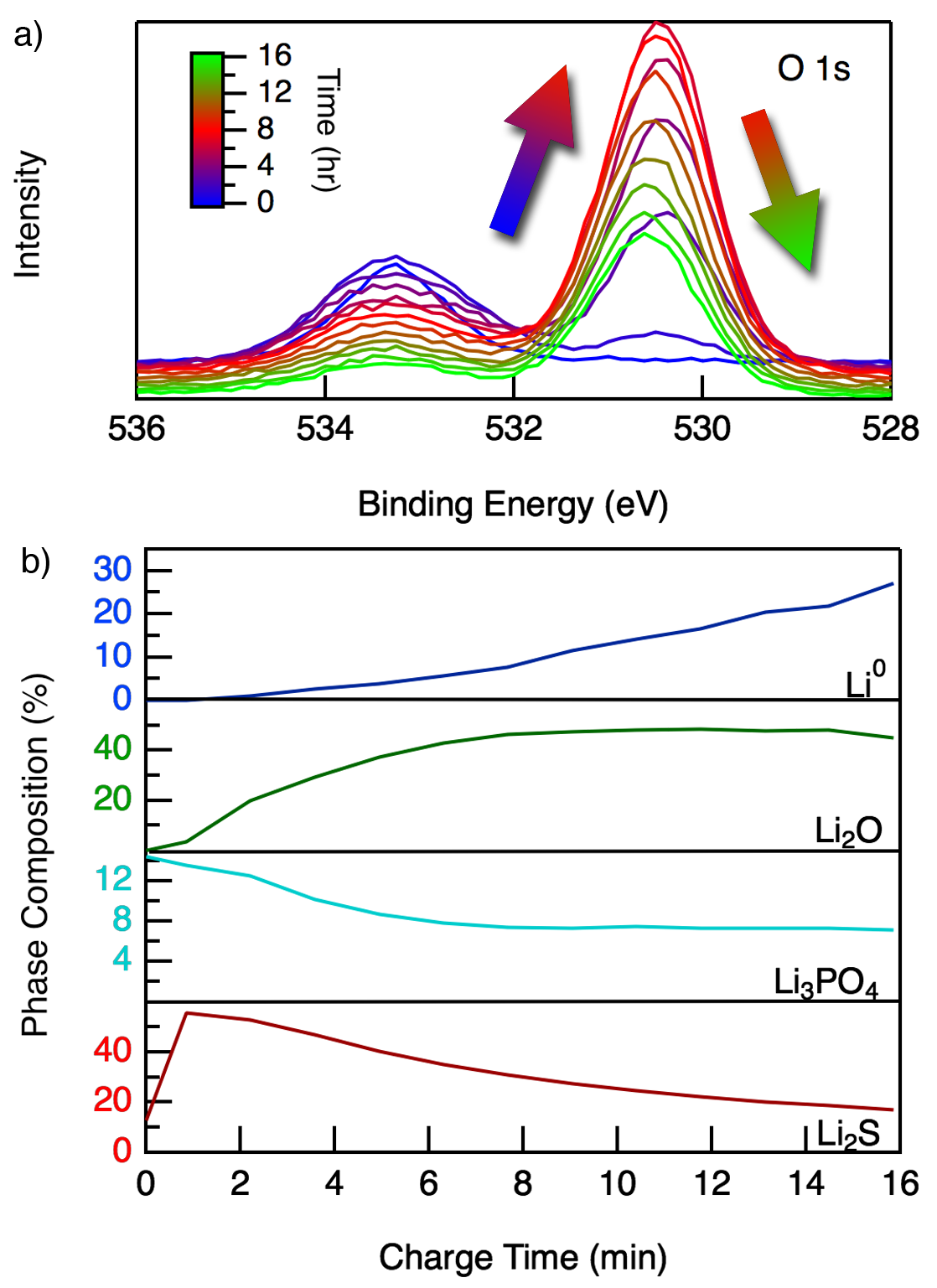


Supplementary Figure 4. a) O 1s XPS spectra from an extended charge cycle, and b) a plot of the phase composition for the extended charge cycle as a function of time for Li_2_O, Li_2_S, Li_3_PO_4_, and Li^0^.


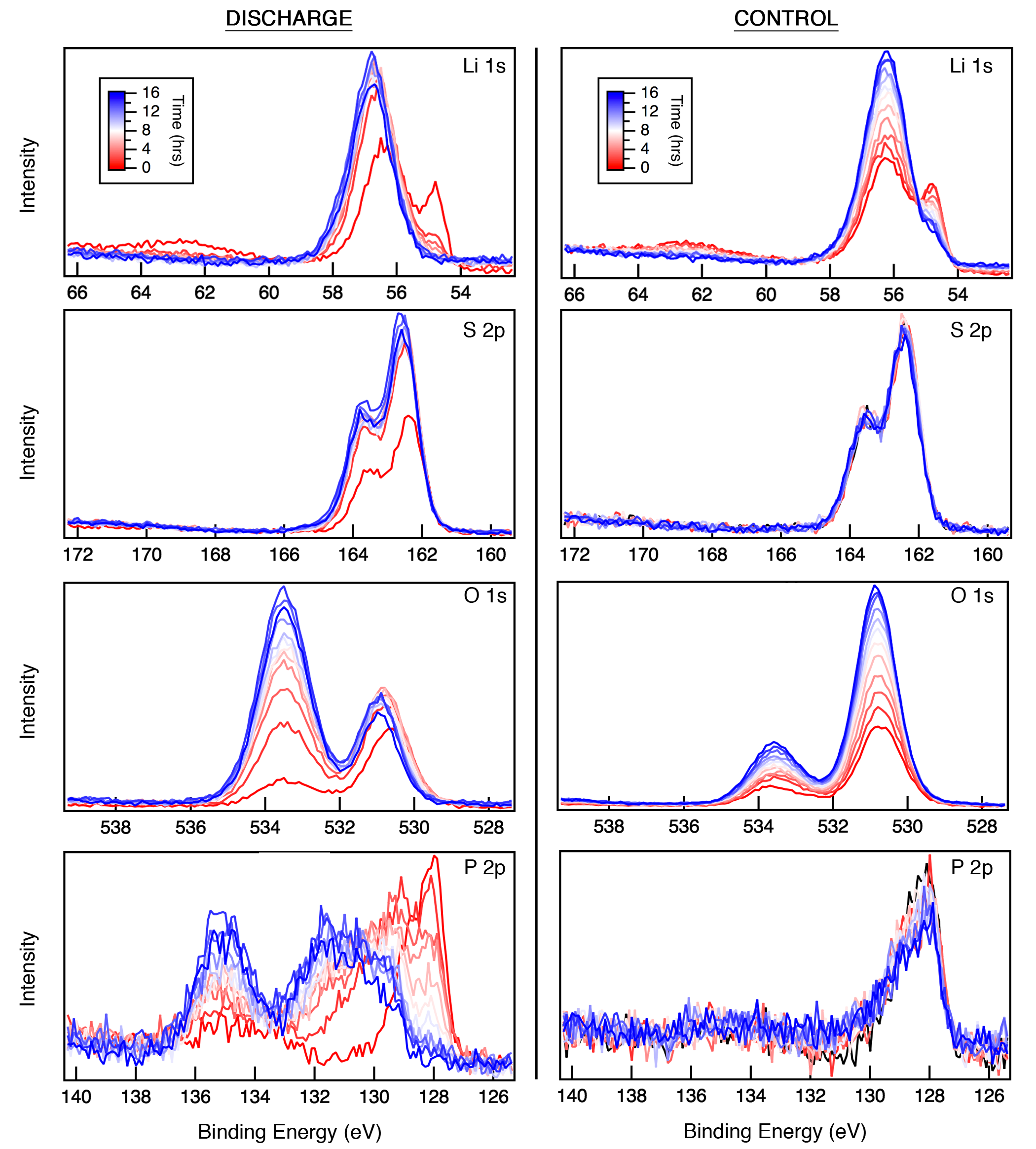


Supplementary Figure 5. Comparison of opXPS spectra acquired from a SEI/LPS/Lifoil sample with the virtual electrode UV light bias over the course a 45 h discharge cycle to those from a control with no UV light bias.


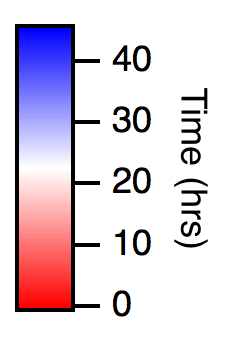

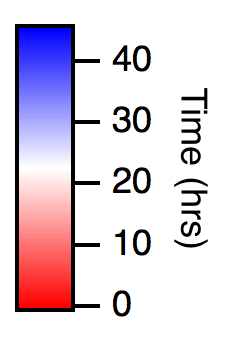

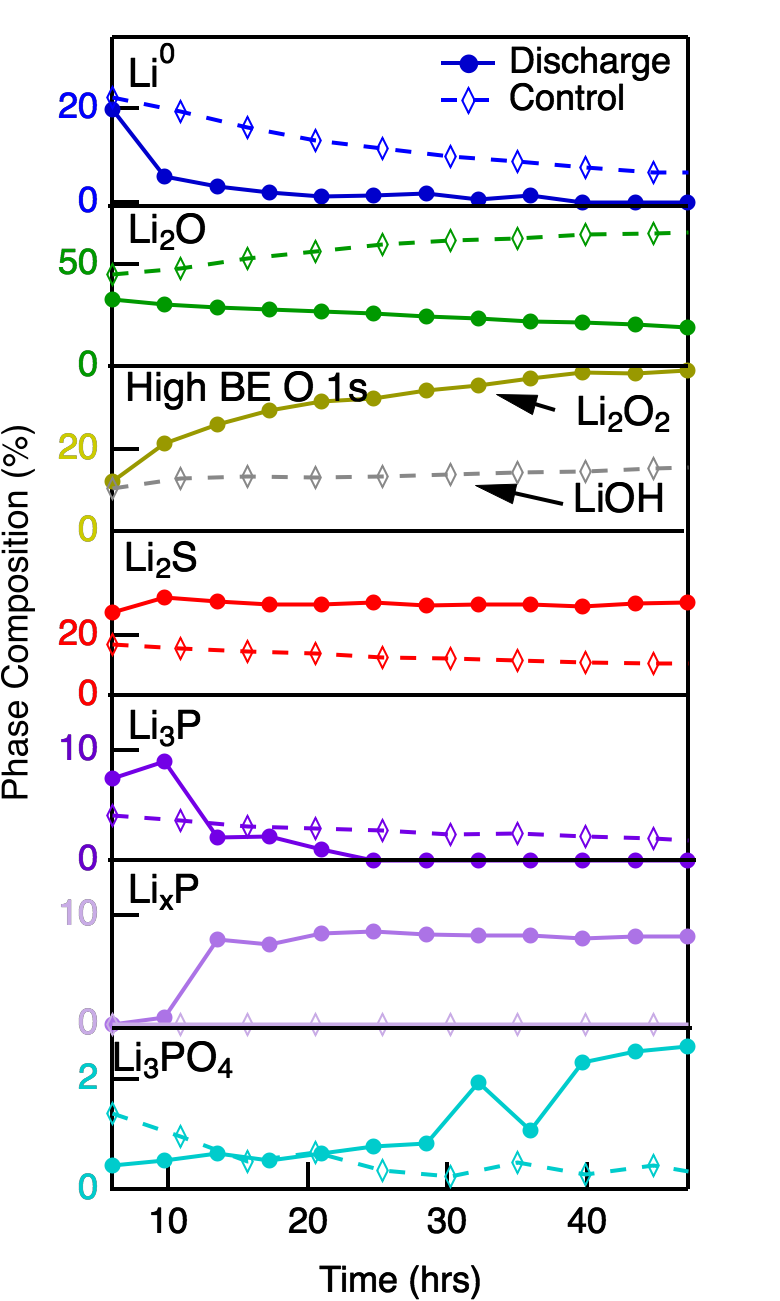


Supplementary Figure 6. Comparison of SEI phase-composition evolution for the operando UV light-biased sample vs. the control sample in Supplementary Figure 5.


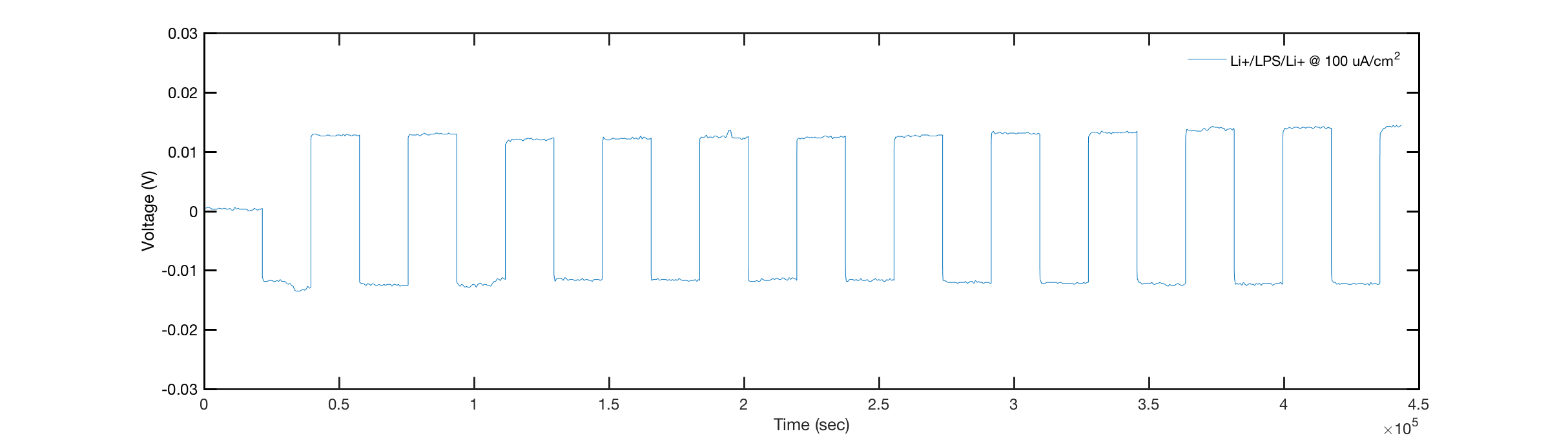


Supplementary Figure 7. Li/LPS/Li symmetric coin-cell data cycled at a similar current density to the opXPS experiments described in the main text. The voltage (cell polarization) recorded from this experiment (125 mV) matches well with the cell polarization values determined from the opXPS BE shifts shown in main text, Fig. 6.


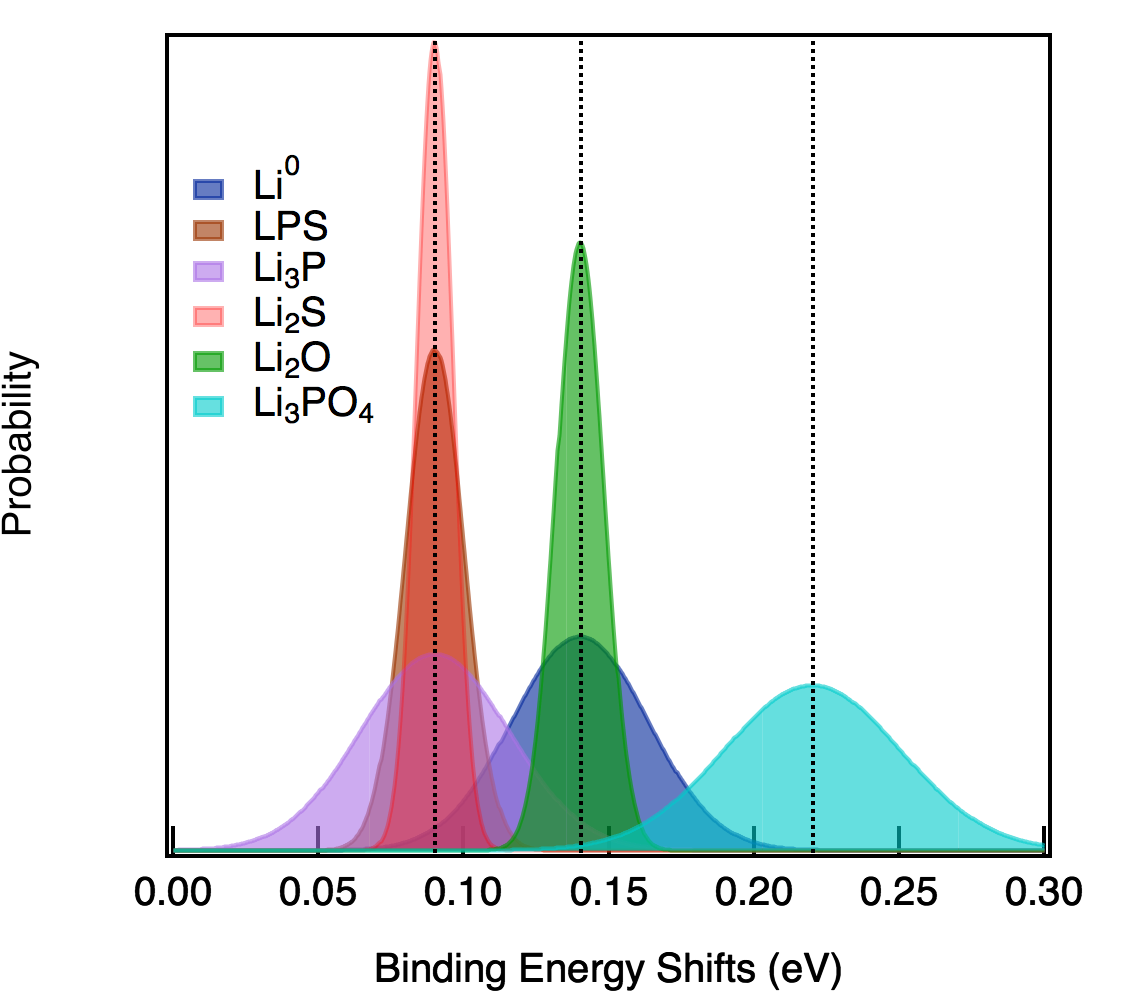


Supplementary Figure 8. Plots of probability distributions vs. BE shifts associated with cell polarizations across each of the observed SEI phases (cf. Fig. 5 in main text). The widths of the Gaussian probability distributions represent calculated uncertainties listed in Supplementary Table 2.


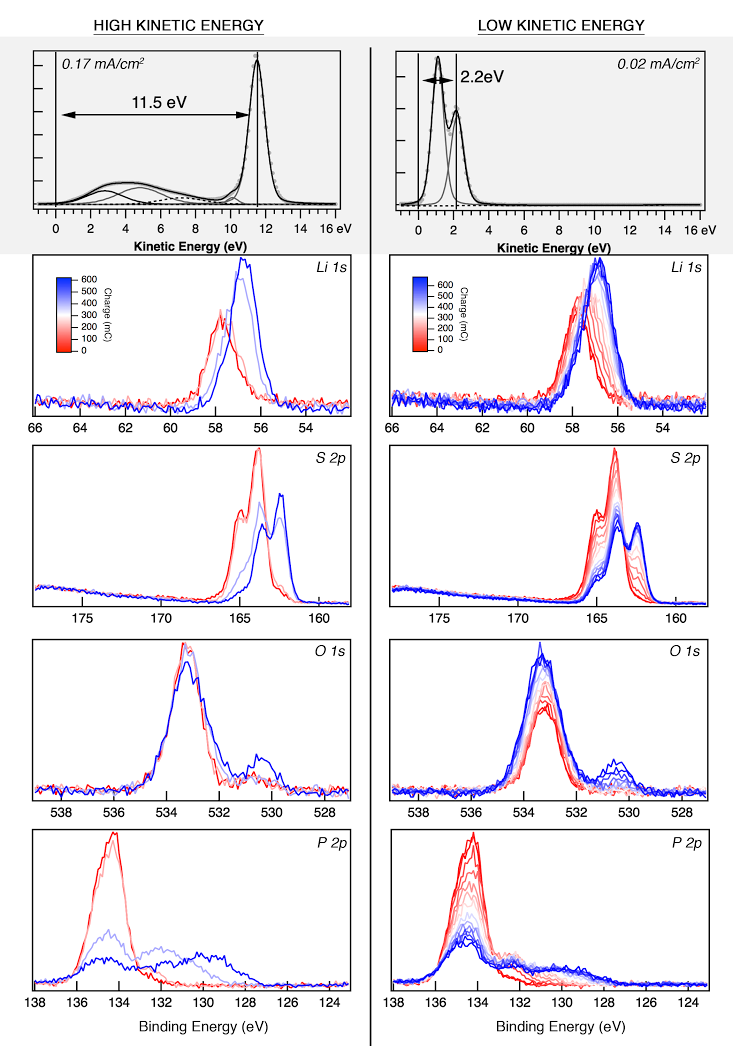


**Supplementary Figure 9.** Comparison of SEI evolution under different ‘virtual electrode’ electron kinetic energies. Current density for the high electron kinetic energy case is about 10 times higher than for the low-energy case. For equivalent amounts of total charge, Q (~600 mC), nearly identical SEI phases are present in both cases.

**Supplementary Table 1.** Fitting constraints used to extract phase composition of the dynamic SEI.

|  | **Fit Constraints** | | | |
| --- | --- | --- | --- | --- |
|  | **Core Level** | **Charge** | **Discharge** | **ΔBE (±.1)** |
| **Li_2_S** | Li 1s | 56.4-56.6 | 56.7-57.0 | 105.8 |
|  | S 2p | 162.2-162.4 | 162.5-162.7 |  |
| **Li_3-x_P** | Li 1s | 57.0-57.5 | 57.1-57.6 | 74-71 |
|  | P 2p | 132-129 | 129-132 |  |
| **Li_3_P** | Li 1s | 57.0-57.5 | 57.1-57.6 | 70.9 |
|  | P 2p | 128.0-128.2 | 128.1-128.3 |  |
| **Li_2_O** | Li 1s | 55.7-55.9 | 55.8-56.0 | 474.8 |
|  | O 1s | 530.4-530.6 | 530.7-530.9 |  |
| **Li_2_O_2_** | Li 1s | None Observed | 56.5-56.8 | 476.7 |
|  | O 1s |  | 533.4-533.6 |  |
| **Li_3_PO_4_** | Li 1s | 56.3-56.5 | 56.5-56.7 | 78.0 398.4 476.4 |
|  | P 2p | 134.4-134.6 | 134.6-134.8 |  |
|  | O 1s | 532.9-533.1 | 533.1-533.3 |  |
| **Li_3_PS_4-x_O_x_** | Li 1s | 57.7-57.9 | None Observed |  |
|  | P 2p | 134.2-134.3 |  |  |
|  | S 2p | 163.7-163.9 |  |  |
|  | O 1s | 533.2-533.3 |  |  |
| **Li^0^** | Li 1s | 54.7-54.9 | 54.8-55.0 |  |
| **-OH** | O 1s | 533.5-533.8 | 533.7-533.9 |  |

**Supplementary Table 2:** Summary of opXPS binding energy uncertainties extracted from curve-fitting standard deviations (σ) used to calculate overpotentials of individual SEI phases.

|  | **Biased Charging** | | | **Unbiased** | | | **Difference** | |
| --- | --- | --- | --- | --- | --- | --- | --- | --- |
|  | **Peak Pos. (eV)** | **Std. dev., σ**  **(eV)** | **unc.,** ±**2σ**  **(eV)** | **Peak Pos. (eV)** | **Std. dev., σ**  **(eV)** | **unc.,** ±**2σ**  **(eV)** | **ΔBE (eV)** | **ΔBE unc. (eV)** |
| **O 1s (Low BE)** | 530.54 | 0.008 | 0.01 | 530.68 | 0.003 | 0.01 | 0.14 | 0.02 |
| **O 1s (High BE)** | 533.19 | 0.030 | 0.05 | 533.41 | 0.013 | 0.02 | 0.22 | 0.05 |
| **P 2p** | 128.01 | 0.025 | 0.04 | 128.10 | 0.017 | 0.03 | 0.09 | 0.05 |
| **Li 1s** | 54.74 | 0.023 | 0.04 | 54.88 | 0.009 | 0.02 | 0.14 | 0.05 |
| **S 2p** | 162.34 | 0.006 | 0.01 | 162.43 | 0.003 | 0.01 | 0.09 | 0.01 |
| **S 2p (LPS)** | 163.88 | 0.010 | 0.01 | 163.97 | 0.008 | 0.02 | 0.09 | 0.02 |

**Supplementary Note 1**

Electrochemical impedance spectroscopy (EIS) data sets acquired during the first three cycles of a Cu_foil_/LPS/Li_foil_ coin cell are summarized Supplementary Figure 1. These results demonstrate that the SEI continues to evolve during cycling. Fitting the component associated with the bulk of the electrolyte reveals the conductivity values for the LPS samples to be 1.61 mS/cm during first half cycle, 1.41 mS/cm after the second half cycle and 1.18 mS/cm after three full cycles. These results are summarized in the legend of Supplementary Figure 1.

**Supplementary Note 2**

Supplementary Table 1 shows the fitting parameters used to decompose individual core level spectra into individual phase components. In addition to the BE parameters shown in this table, the sensitivity-factor-adjusted peak areas for core levels associated with particular phases were also constrained. For example when fitting the Li_2_S phase of the SEI, the relative intensities were constrained such that Li_2_S component in the Li 1s core level was twice that of the Li_2_S component in the S 2p. These intensity ratios were constrained to +/- 5% of ideal values. In addition to BE positions and intensities, the BE separation between different core levels of the same SEI component were also monitored and required to be within 0.5% of the value shown in right most column of the table.

**Supplementary Note 3**

The data in Supplementary Figure 2 demonstrate that the SEI interfacial region in a traditionally cycled LPS sample (Cu_foil_/LPS/Li_foil_ coin cell) exhibits strong similarities to the opXPS-cycled LPS/Li_foil_ sample. Only two significant differences are evident: 1) Cu_2_S is observed in the S 2p for the coin cell due to an additional reaction between the LPS and Cu electrode; and 2) less Li_3_P is observed in the coin cell cycled sample. The latter effect is likely due to disassembly related artifacts including reactions with oxygen or moisture that form C and O containing phases as well as Li_3_PO_4_.

**Supplementary Note 4**

Due to the extreme reactivity of Li metal and the unavoidable presence of trace H_2_O and other gases in the XPS chamber (typically < 5 × 10^-10^ Torr), it is expected that small amounts of oxygen-containing species form continuously on the exposed Li^0^ layer. On the other hand, we can estimate the minimum time for one complete monolayer (ML) of O-containing phases at the Li^0^ surface as follows. For 5×10^-10^ Torr, using H_2_O as an example, we calculate an impingement rate ~6×10^11^ molecules•cm^-2^•s^-1^. Assuming unity sticking coefficient, one full ML (~1.3×10^15^ Li sites•cm^-2^) of –O or –OH will form in ~1 h. For the opXPS experimental conditions tested, we estimate the Li^+^ current density to be > 0.1 mA cm^-2^, corresponding to 6×10^14^ Li^+^ cm^-2^ s^-1^, or ~0.5 ML s^-1^. Therefore, at typical opXPS experimental conditions, the Li^+^ arrival rate at the surface is ~1000 times larger than that of residual gas molecules. On this basis we conclude that Li^0^ plating occurs faster than conversion to O-containing phases by reactions with trace gases. For reference, the spectra of a ‘pristine’ Li metal surface (obtained by extended Ar^+^-ion sputtering of a Li foil sample) is shown in Supplementary Figure 3.

**Supplementary Note 5**

To demonstrate that oxygen observed in SEI phases originates predominantly within the LPS solid electrolyte rather than from trace residual gases in the XPS analysis chamber, an LPS/Li_foil_ sample was charged for 16 h. The data reveals that after ~10 h the oxygen content in the sample reaches a maximum and begins to attenuate as Li metal is plated above the Li_2_0 layer (Supplementary Figure 4a); and similarly, the Li_3_PO_4_ and Li_2_S phases attenuate (Supplementary Figure 4b). As discussed above, the observed quantities of O-containing phases in the SEI cannot be accounted for by the reactions with residual gases, because the impingement rates of the latter are far too low. In addition, the appearance and continued growth Li^0^ is further evidence that the arrival rate of Li^+^ at the surface due to the *operando* current bias is substantially larger than the reaction rate of exposed Li^0^ with trace gases. If this were not the case, the Li^0^ feature would never appear, and there would be no mechanism to explain the attenuation of Li_2_O by Li^0^.

**Supplementary Note 6**

Control experiments were performed to assess the relative contributions of residual gas contamination to compositional and chemical changes observed during the opXPS discharge half cycle. In the control experiment Li metal was brought to the surface via the virtual electrode, and subsequently XPS measurements, identical to those performed in the discharge cycle, were used to measure changes on the surface under similar ambient vacuum conditions (~6x10^-10^ Torr), but without the UV-light light bias (Supplementary Figure 5). By comparing these data sets, it is clear that the changes observed in the O 1s core level during the discharge process are distinct from those caused by exposure to residual gases, and resulted in different effects. In the control experiment both the Li_2_O peak and the low-BE Li-O phases grew slightly over the 45 h experiment, in roughly equal proportions, an effect that has been documented previously.^1,2^ However, this is very different than the discharge experiment, where the Li_2_O remained nearly constant and the low-BE peak assigned to Li_2_O_2_ increased substantially. Therefore, we conclude that the changes demonstrated in main text Figs. 3 and 4 (and in Supplementary Figure 5) must be electrochemical effects that occur as a result of Li^+^-ion migration driven by the UV LED light source. Furthermore, while it is almost certain that some of the Li_2_O signal comes from metallic Li reacting with the UHV environment, we conclude that the majority of oxygen observed in the SEI originates within the LPS itself. This is supported by evidence, which shows that during the extended charge cycle (16 h) Li^0^ attenuates the Li_2_O signal (Supplementary Figure 4). This would not occur if the Li_2_O phase formed only at the electrode/UHV interface. In addition, the control experiment shows no significant changes in either the Li_2_S or Li_3_P features, indicating that these phases are chemically stable over at least the duration of the experiment, and that changes driven by XPS photocurrents must be small relative to those driven by UV light bias. A comparison of phase compositions for these two experiments are plotted in Supplementary Figure 6.

**Supplementary Note 7**

For the control sample profile data shown in Supplementary Figure 6, it is likely that the slight increase associated with the high-BE features of the O 1s is due to -OH bonding (rather than Li_3_PO_4_ or Li_2_O_2_). By comparison, during discharge (*operando* UV light bias case) a dramatic increase in intensity of the high-BE O 1s feature, combined with a slight BE shift, is consistent with assigning the majority of this peak to Li_2_O_2_, again demonstrating distinct differences between the control and the discharge cases.

**Supplementary Note 8**

A symmetric Li_foil_/LPS/Li_foil_ coin cell was assembled and cycled at the same current density (0.17 mA/cm^2^) estimated for the opXPS measurements performed on the LPS/Li_foil_ described in the main text. The net cell polarization observed for the symmetric coin cell closely matches values extracted from the opXPS measurements, as summarized in Supplementary Figure 7.

**Supplementary Note 9**

To assess the reliability of the chemically resolved overpotential values measured in this study, special attention was devoted XPS measurement uncertainties and error propagation. Supplementary Table 2 shows the uncertainty measurements calculated for each cell polarization measurement. Standard deviations (σ) are extracted from curve fitting of the XPS core levels, and from these BE uncertainties are set to ±2σ. From these values, standard error-propagation methods are used to find uncertainties for subsidiary values, i.e. those associated with BE shifts between the *operando* biased and unbiased cases. The normal (Gaussian) probability distributions for BE shifts associated with *operando* cell polarization were calculated according to Eq. SI1,

$y=\frac{1}{\sqrt{2\pi\sigma^{2}}}e^{\frac{-{(x-\mu)}^{2}}{2\sigma^{2}}}$ (SI1)

where $\sigma$ is the standard deviation and $\mu$ is the measured BE value. The resultant probability distributions are plotted in Supplementary Figure 8.

**Supplementary Note 10**

From these distributions it is now possible to calculate the relative probability that any particular SEI phase was at a higher polarization than another during the *operando* biasing. These relative probabilities can be calculated according to Eq. SI2 as the product of the integrals of the individual probability distributions over complementary ranges:

$P\left( PhaseA>PhaseB \right)=\int_{-\infty}^{z} P(phaseA)*\int_{z}^{\infty} P(phaseB)$ , (SI2)

where z is the delimiting BE value.

**Supplementary Note 11**

To demonstrate that the effects seen during the 'fast' charge cycle are not due to the high electron kinetic energy (KE = 11.5 eV, 0.17 mA/cm^2^), a charge cycle was completed using lower KE electrons (2.2 eV, 0.02 mA/cm^2^), as shown in Supplementary Figure 3. These results demonstrate that the extent of SEI formation is independent of the virtual-electrode electron KE, and depends only on the net charge that has passed. These experiments were completed under nearly identical conditions in the analysis chamber (< 10^-9^ torr). For the data shown in the main text, the higher electron KE conditions were selected to maximize available current density and reduce acquisition times for the experiment.

**Supplementary References**

(1) Zavadil, K. R.; Armstrong, N. R. *Surf. Sci.* **1990**, *230* (1–3), 61–73.

(2) Wang, K.; Ross Jr., P. N.; Kong, F.; McLarnon, F. *J. Electrochem. Soc.* **1996**, *143* (2), 422–428.
