# Supplementary figures and images for: Operando X-ray photoelectron spectroscopy of solid electrolyte interphase formation and evolution in Li2S-P2S5 solid-state electrolytes
Source: Nat Commun. 2018 Jun 27;9:2490. doi: 10.1038/s41467-018-04762-z (PMC6021442; doi:10.1038/s41467-018-04762-z)

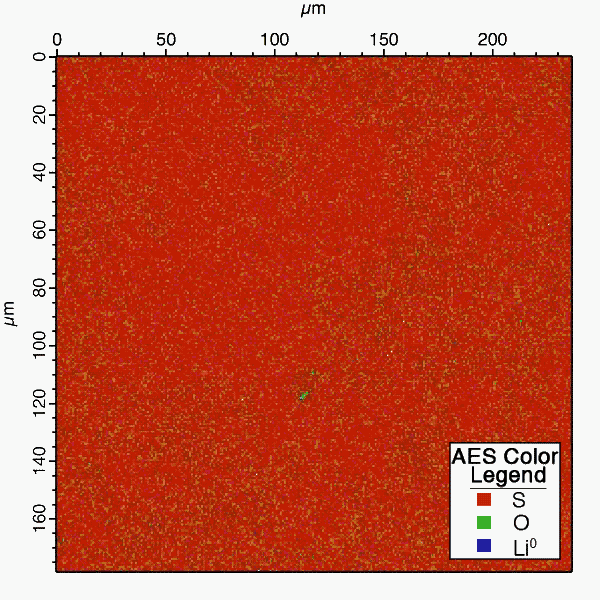

Supplement: Supplementary file 2 — Supplementary Movie 1 [file 41467_2018_4762_MOESM2_ESM.gif]
