# Supplementary material for: Operando X-ray photoelectron spectroscopy of solid electrolyte interphase formation and evolution in Li2S-P2S5 solid-state electrolytes
Source: Nat Commun. 2018 Jun 27;9:2490. doi: 10.1038/s41467-018-04762-z (PMC6021442; doi:10.1038/s41467-018-04762-z)
Supplement: Supplementary file 3 — Description of Additional Supplementary Files [file 41467_2018_4762_MOESM3_ESM.pdf]

Description of Additional Supplementary Files

File Name: Supplementary Movie 1

Description: A sequence of in situ Auger Electron Spectroscopy (AES) images shows the compositional inhomogeneity during a virtual electrode charge step. Red represents sulfur, Green represents oxygen, and blue represents lithium metal. Lithium metal can be differentiated from other Li-containing phases using AES.
